# *w*Mel limits zika and chikungunya virus infection in a Singapore *Wolbachia*-introgressed *Ae*. *aegypti* strain, *w*Mel-Sg

**DOI:** 10.1371/journal.pntd.0005496

**Published:** 2017-05-19

**Authors:** Cheong Huat Tan, PeiSze Jeslyn Wong, Meizhi Irene LI, HuiTing Yang, Lee Ching Ng, Scott Leslie O’Neill

**Affiliations:** 1 Environmental Health Institute, National Environment Agency, Singapore, Singapore; 2 School of Biological Sciences, Monash University, Melbourne, Australia; 3 School of Biological Sciences, Nanyang Technological Institute, Singapore, Singapore; Centers for Disease Control and Prevention, UNITED STATES

## Abstract

**Background:**

Zika (ZIKV) and Chikungunya (CHIKV) viruses are emerging Aedes-borne viruses that are spreading outside their known geographic range and causing wide-scale epidemics. It has been reported that these viruses can be transmitted efficiently by *Ae*. *aegypti*. Recent studies have shown that *Ae*. *aegypti* when transinfected with certain *Wolbachia* strains shows a reduced replication and dissemination of dengue (DENV), Chikungunya (CHIKV), and Yellow Fever (YFV) viruses. The aim of this study was to determine whether the *w*Mel strain of *Wolbachia* introgressed onto a Singapore *Ae*. *aegypti* genetic background was able to limit ZIKV and CHIKV infection in the mosquito.

**Methodology/Principal findings:**

Five to seven-day old mosquitoes either infected or uninfected with *w*Mel Wolbachia were orally infected with a Ugandan strain of ZIKV and several outbreak strains of CHIKV. The midgut and salivary glands of each mosquito were sampled at days 6, 9 and 13 days post infectious blood meal to determine midgut infection and salivary glands dissemination rates, respectively. In general, all wild type *Ae*. *aegypti* were found to have high ZIKV and CHIKV infections in their midguts and salivary glands, across all sampling days, compared to *Wolbachia* infected counterparts. Median viral titre for all viruses in *Wolbachia* infected mosquitoes were significantly lower across all time points when compared to wild type mosquitoes. Most significantly, all but two and one of the *w*Mel infected mosquitoes had no detectable ZIKV and CHIKV, respectively, in their salivary glands at 14 days post-infectious blood meal.

**Conclusions:**

Our results showed that wMel limits both ZIKV and CHIKV infection when introgressed into a Singapore *Ae*. *aegypti* genetic background. These results also strongly suggest that female *Aedes aegypti* carrying *Wolbachia* will have a reduced capacity to transmit ZIKV and CHIKV.

## Author summary

Zika (ZIKV) and Chikungunya (CHIKV) viruses are emerging Aedes-borne viruses that are spreading outside their known geographic range and causing wide-scale epidemics. It has been shown that these viruses can be transmitted efficiently by Aedes aegypti. Recent studies have shown that Ae. aegypti when transinfected with certain Wolbachia strains shows a reduced replication and dissemination of several arboviruses, including dengue and yellow fever viruses. This study examines the effect of the wMel strain of Wolbachia in Singapore’s Ae. aegypti on susceptibility of these mosquitoes to ZIKV and CHIKV and finds that there is very strong inhibition of replication and dissemination of both viruses in mosquitoes that contain Wolbachia. Results from this study strongly suggest that female Ae. aegypti carrying Wolbachia will have a reduced capacity to transmit ZIKV and CHIKV. This indicates that establishment of Wolbachia in Ae. aegypti populations should reduce transmission of these viruses and presents a potential control measure in this setting.

## Introduction

Vector-borne diseases are leading causes of morbidity and mortality worldwide. Factors such as rapid urbanization, migration, increase in international trade and travel, climate change and pathogens’ plasticity in adapting to new hosts and vectors have contributed to the spread of emerging and re-emerging mosquito-borne diseases worldwide. The propensity of mosquito-borne diseases to spread outside their geographic range and cause wide-scale epidemics has clearly been demonstrated by the recent zika outbreaks in French Polynesia and South America and the recent chikungunya outbreak in La Reunion Island. During the outbreak in La Reunion Island, 37% of the population was infected by chikungunya virus (CHIKV)[1] while it was estimated that 73% of the local residents at Yap Island were infected with Zika virus (ZIKV)[2]. Most recently, the Brazil’s Ministry of Health has estimated that up to 1.3 million people were infected with ZIKV (ECDPC, 2015). Whilst CHIKV has been reported to be circulating in South East Asia since 1958 [3] Singapore did not report local transmission until 2008 [4]. During 2008–2009 period, two independent outbreaks occurred in the country, the first localized outbreak in January-February 2008 and an island wide outbreak that started in June 2008. Both outbreaks were caused by CHIKV strains of the East, Central and South African (ECSA) genotype [5–7]. However, the ECSA strains involved in the first and second outbreaks differed genetically as well as phenotypically as the second outbreak strains possessed the E1-A226V substitution, which increased its transmissibility by *Aedes albopictus*[7]. CHIKV re-emerged in 2013 when a newly-introduced ECSA strain caused an island wide outbreak.

Control of these mosquito-transmitted diseases is difficult and hampered by lack of effective tools beyond classical insecticide-based mosquito control and environmental management. In recent years some novel approaches to vector control are being developed that show promise for disease control. One of these approaches is through the use of *Wolbachia*-carrying mosquitoes. *Wolbachia* is a maternally-inherited endosymbiotic bacterium present in 70% of insect species and is known to affect various reproduction manipulations in its hosts[8, 9]. The bacteria are found in several medically and veterinary important mosquito species, but was absent in the major dengue vector *Ae*. *aegypti* [10]. At present, *Wolbachia* is receiving considerable global attention for its potential as a biological control tool against dengue and other mosquito-borne diseases. *Wolbachia*-based strategies include population replacement strategy and the incompatible insect technique (IIT).

Recent studies have shown that *Ae*. *aegypti* transinfected with *Wolbachia* are able to reduce the replication and transmission of DENV and CHIKV[11–14]. The aim of the control strategy is to introduce *Wolbachia* into wild *Ae*. *aegypti* populations by deliberately releasing both males and females *Wolbachia*-infected *Ae*. *aegypt* [15, 16]. The ability of *Wolbachia* to manipulate mosquito reproduction using cytoplasmic incompatibility (CI) and its mode of transmission have provided the bacterium with a reproductive advantage, thus ensuring its spread into uninfected wild *Ae*. *aegypti* populations [9, 16].

In this study, we assess whether the *Wolbachia* strain, *w*Mel, is able to limit East African ZIKV and CHIKV infections and dissemination in Singapore *Ae*. *aegypti* under local environmental conditions.

## Materials and methods

### Mosquitoes

Two different strains of *Ae*. *aegypti* were used in the experiments, a local field strain (WT) and a *Wolbachia*-infected strain (*w*Mel-Sg) that through a series of backcrosses was bred to contain >98% WT genetic background. The local *Ae*. *aegypti* strain used for the experiments, was established from larvae collected from residential premises across the island during routine inspections by enforcement officers of the National Environment Agency, Singapore. Mosquitoes were allowed to emerge and were maintained under standard insectary conditions at 27± 1°C and 75–80% relative humidity (RH), with a photoperiod of 12h:12h light:dark (L:D) cycles. F_0_ mosquitoes were allowed to mate randomly and were fed with commercially obtained swine’s blood using a Hemotek membrane feeding system (Discovery Workshops, UK). F_1_ eggs were allowed to hatch in de-chlorinated water. Two hundred fifty larvae were reared in enamel pans measuring 25cm x 30cm x 9cm containing approximately 2 L of water and fed with Plecomin fish food (Tetra, Germany). Pupae were placed inside 30cm x 30cm x 30cm cages and allowed to emerge into adults. The F_3_ generations were used in the feeding experiments.

To generate a local *Wolbachia*-infected *Ae*. *aegypti* strain (wMel-Sg), 300 female *w*Mel-infected *Ae*. *aegypti* (Cairns strain), obtained from the O’Neill Laboratory in Monash University, Australia, were backcrossed with equal numbers of local wild-type male *Ae*. *aegypti* (F_3_). In the next generation, the same number of the resulting hybrid females were mated with an equal number of wild-type males (F_3_). This backcrossing was repeated for six generations following the rearing methods for wild-type mosquitoes described above. After six generations of backcrossing the colony was closed and the subsequent generation used in the study.

The F3 mosquitoes used in the backcrossing experiments and competence study were derived from the same F1 parental lineage. All mosquitoes were starved for at least 24 hours prior to the infectious feed.

### Virus strains

The Ugandan MR766 ZIKV strain used in the study was obtained from the American Type Culture Collection (ATCC, USA). This virus was originally isolated in 1947, from the blood of a sentinel rhesus monkey. The CHIKV strains used in this study were isolated in Singapore. All CHIKV strains belong to the ECSA genotype. The EHI0067Y08 (GenBank: EU441882) strain was isolated from a patient during the first outbreak of chikungunya in Singapore in January – February 2008 [4], while EHIKJ71albY08 and EHI66SGKalbY13strains were isolated from *Ae*. *albopictus* during the subsequent outbreaks in 2008[17] and 2013 (GenBank: KX925219), respectively. EHI0067Y08 did not possess the alanine to valine mutation at amino acid position 226 of the membrane fusion glycoprotein E1 gene, but was present in both EHIKJ71albY08 and EHI66SGKalbY13. All viruses used in the oral infection of mosquitoes have been passaged three times in Vero cells (ATCC, USA) prior to the infectious feed.

### Vector competence experiments

Fresh viruses, mixed with 100% swine-packed Red Blood Cells, were used to infect five to seven-day old female mosquitoes to compare the infection and dissemination rates of ZIKV and CHIKVs in wMel-Sg and WT mosquitoes. Final concentration of ZIKV in blood meal was 7.34 Log_10_ tissue culture infectious dose_50_ per mL (Log_10_TCID_50_/mL); and those of CHIKVs, EHI0067Y08, EHIKJ71albY08 and EHI66SGKalbY13 strains were 7.14, 6.67 and 6.81 Log_10_TCID_50_/mL, respectively. Adenosine Triphosphate (Fermentas, USA) at a final concentration of 3mM was added to the infectious blood meal as a phagostimulant. For each of the infectious blood meals, 120 females were placed in 0.5L ca. containers and were allowed to feed on swine’s blood mixed with fresh virus suspension. After thirty minutes of feeding, all mosquitoes were cold anesthetized on ice and 20 to 25 fully engorged females were transferred to a 300 ml ca. paper cups with nettings on top. The engorged females were maintained in an environmental chamber (Sanyo, Japan) set at cyclical temperatures between 29°C to 31°C and 70–80% RH with a photoperiod of 12hL:12hD cycles and were provided with 10% sugar solution. The conditions set in the environmental chamber simulates that of indoor conditions in Singapore which was determined by placing data loggers inside naturally ventilated living rooms of eight homes randomly distributed across the island (Appendix 1). To determine viral midgut infection and salivary gland dissemination rates, at least ten wMel-Sg and WT mosquitoes were sampled at days 6, 9 and 13 post-infectious (p.i.) blood meals. All experiments were carried out inside an arthropod containment level 2 facility.

### Processing of mosquitoes

The midgut and salivary glands of each mosquito were processed as previously described[18, 19]. Briefly, the midgut and both pairs of salivary glands were homogenized using three millimetre stainless grinding balls (Retsch, Germany) in a MM301 mixer mill (Retsch, Germany) set at a frequency of 12/sec for 1 min. The supernatant of the homogenates was used either for viral titration or real-time PCR assays.

ZIKV midgut and salivary glands titres were determined using the tissue culture infectious dose 50 (TCID50) assay, an endpoint dilution technique, using Vero cells[20]. Briefly, 100 mL of 10-fold serial dilutions from each homogenate sample were titrated in 96-well microtitre plates and incubated with Vero cells (ATCC, USA) inside an incubator set at 37C and 5% CO2. At the end of a seven-day incubation period, the cells were examined microscopically for viral-induced cytopathic effect (CPE). A well was scored positive if any CPE was observed. All virus titres were expressed as Log_10_TCID_50_/mL.

CHIKV salivary glands titre was determined using a TCID50 assay, while CHIKV midgut viral load was determined by a one-step RT-qPCR assay targeting the non-structural protein 1 (*nsP1*) gene of CHIKV[21], using Rotor-Gene Q (Qiagen, Germany) PCR machine. Briefly, total RNA was isolated from a mosquito’s midgut using the QIAamp Viral Mini Kit (Qiagen, Germany) following manufacturer’s recommendation. Primers used were Chik nsP1F: 5’-TAGAGCAGGAAATTGATCCC-3’ and Chik nsP1R: 5’- CTTTAATCGCCTGGTGGTAT-3’. The PCR reactions were carried out using the Quantitech SYBR Green RT-PCR kit (Qiagen, Germany) following manufacturer’s recommendation. Amplification of the target gene from each individual mosquito midgut was compared against a standard curve generated from 10-fold serial dilutions of RNA standard. Viral RNA copies below 10 were considered negative.

### Data analysis

The midgut infection and salivary gland dissemination rates at each time point were calculated by dividing the number of infected midguts and salivary glands, respectively, by the total number of midguts and salivary glands sampled. Differences in midgut and salivary gland infection rates were analysed using paired Fisher’s exact tests. Kolmogorov-Smirnov tests indicated that the data did not conform to conditions of normality, hence non-parametric analyses were performed. The differences in each of the viruses titre between WT and wMel-Sg mosquitoes were analysed using the Mann-Whitney *U*-tests. All statistical tests were performed using the MedCalc for Windows (MedCalc software, Belgium).

## Results

### *w*Mel limits ZIKV infection and dissemination in *Ae*. *aegypti*

In the first experiment, pairwise comparison of midgut infection and salivary gland dissemination rates showed that *w*Mel-Sg had significantly lower ZIKV infection rate when compared to WT mosquitoes (Table 1). All WT *Ae*. *aegypti* were found to have ZIKV in their midguts and salivary glands, across all sampling days, while fewer *w*Mel-Sg exhibited ZIKV infections in their midguts (71% (n = 14), 53% (n = 15) and 40% (n = 14) at days 6, 9, and 13 p.i., respectively) and nearly all of these mosquitoes had no detectable virus in their salivary glands (7.1% (n = 14), 6.7% (n = 15) and 14% (14) at days 6, 9 and 13 p.i., respectively). Pairwise comparison (Fisher’s Exact Test, *P*<0.05) showed a significantly fewer *w*Mel-Sg *Ae*. *aegypti* were infected ZIKV compared to WT mosquitoes.

**Table 1:** Infection and dissemination rates for ZIKV and CHIKV among WT and *w*Mel-Sg *Ae*. *aegypti* strains at days 6-, 9- and 13-days infectious blood meal.

Virus	Strains	% Infections (N)											
		Day 6 p.i.				Day 9 p.i.				Day 13 p.i.			
		Midgut		Salivary glands		Midgut		Salivary glands		Midgut		Salivary glands	
		WT	wMel-Sg	WT	wMel-Sg	WT	wMel-Sg	WT	wMel-Sg	WT	wMel-Sg	WT	wMel-Sg
ZKV	MR766	100 (10)	71 (14)	100 (10)	7.1 (14)*	100 (10)	53 (15)*	100 (10)	6.7 (15)*	100 (10)	40 (14)*	100 (10)	14 (14)*
CHKV	EHI0067Y08	100 (10)	20 (10)*	100 (10)	0 (10)*	100 (10)	30 (10)*	100 (10)	0 (10)*	100 (10)	40 (10)*	100 (10)	0 (10)*
	EHIKJ71albY08	100 (10)	60 (10)	100 (10)	10 (10)*	100 (10)	30 (10)*	100 (10)	0 (10)*	100 (10)	40 (10)*	100 (10)	10 (10)*
	EHI66SGKalbY13	100 (10)	60 (10)	100 (10)	10 (10)*	100 (10)	0 (10)*	100 (10)	0 (10)*	100 (10)	30 (10)*	100 (10)	0 (10)*

Fisher’s Exact Test (*P* value <0.05 are highlighted by*). Pairwise comparison showed a significantly fewer *w*Mel-Sg *Ae*. *aegypti* were infected with CHIKV and ZIKV compared to WT mosquitoes.

**Fig 1:**
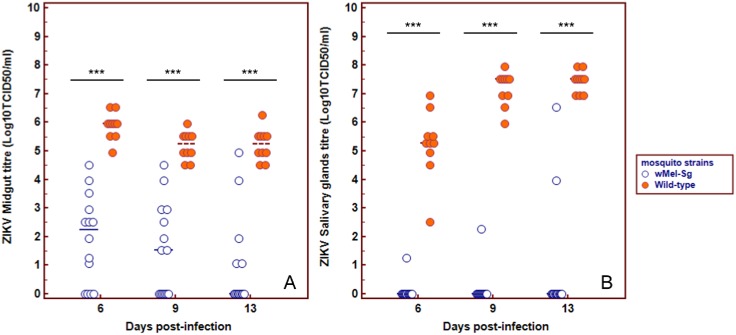
*w*Mel inhibit the dissemination of ZIKV infection in Singapore’s *Ae*. *aegypti*. Titre of ZIKV in midguts and salivary glands of *w*Mel-Sg and WT *Ae*. *aegypti* at days 6, 9 and 13 post-infectious blood meal. ZIKV level is both midguts and salivary glands were determined using viral titration assay and expressed as Log_10_TCI_50_/mL. Bars denote median viral titres. *** denotes significant difference at P<0.05 by Mann-Whitney test. Each point represents an individual midgut/salivary glands.

When mosquitoes were infected with ZIKV the midgut titres of wMel-Sg were significantly lower compared to the WT mosquitoes (Fig 1). Similarly, the four wMel-Sg with detectable virus in their salivary glands, had lower ZIKV titres when compared to the salivary gland viral titre in WT mosquitoes.

### wMel reduces CHIKV infection and blocks virus dissemination in *Ae*. *aegypti*

In the second experiment, we determined the ability of the *w*Mel strain to block the dissemination of three CHIKV strains in *Ae*. *aegypti*. Irrespective of the CHIKV strains used to orally infect both *Ae*. *aegypti* strains, a significantly lower number of *w*Mel-Sg had infections in their midguts and salivary glands when compared to the WT mosquitoes (Table 1). The midguts and salivary glands of all WT mosquitoes tested were found to be infected with CHIKV, across all time-points, except for one sample at day 6 p.i. (Table 1). On the other hand, no more than 60% of the *w*Mel-Sg sampled at different days p.i. have detectable CHIKV RNA in their midguts. Of these, only three of the *w*Mel-Sg has detectable CHIKV in their salivary glands, two (EHIKJ71albY08 and EHI66SGKalbY13) at day 6 p.i. and one (KJ71) at day 13 p.i. None of the *w*Mel-Sg sampled at day 9 p.i. had detectable infectious CHIKV in their salivary glands. In general, pairwise comparison (Fisher’s Exact Test, *P*<0.05) showed a significantly fewer *w*Mel-Sg *Ae*. *aegypti* were infected CHIKV compared to WT mosquitoes, especially at day 9 and 13 pi.

**Fig 2:**
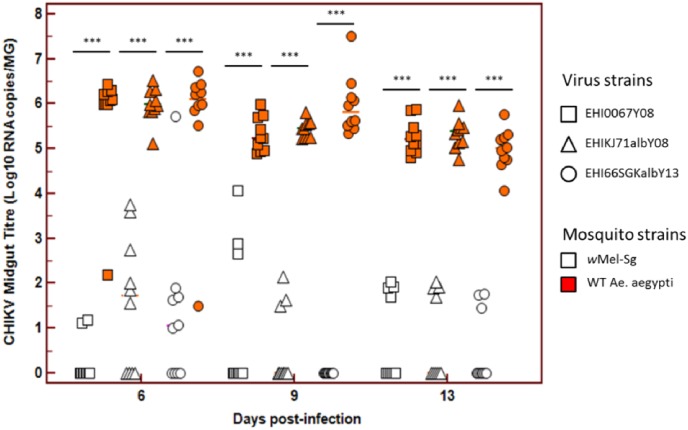
*w*Mel reduces midgut CHIKV infection in Singapore’s *Ae*. *aegypti*. Midgut CHIKV titre in WT and wMel-Sg *Ae*. *aegypti* at days 6, 9 and 13 post-infections. CHIKV levels in midguts were determined using qRT-PCR assay and expressed as Log10 RNA copies/ml. Bars denote median viral titres. *** denotes significant difference at P<0.05 by Mann-Whitney test. Each point represents an individual midgut.

**Fig 3:**
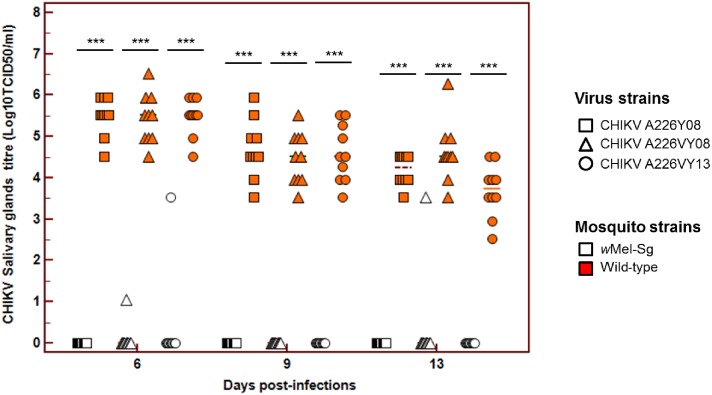
*w*Mel inhibits CHIKV salivary glands dissemination in *Ae*. *aegypti*. Salivary glands CHIKV titre in WT and wMel-Sg *Ae*. *aegypti* at days 6, 9 and 13 post-infections. CHIKV levels in both salivary glands were determined salivary glands using viral titration assay and expressed as Log_10_TCI_50_/mL. Bars denote median viral titres. *** denotes significant difference at P<0.05 by Mann-Whitney test. Each point represents an individual salivary glands.

Overall, *w*Mel-Sg has significantly lower CHIKV titre in its midgut when compared to WT *Ae*. *aegypti* across all time points (Fig 2). Only a small proportion of the salivary glands of *w*Mel-Sg mosquitoes were infected in these mosquitoes, and the CHIKV titres in these organs were also lower compared to the median WT CHIKV titre (Fig 3).

## Discussion

Mosquito-borne arboviruses and parasites pose a continuous and significant public health threat globally. Novel vector control tools such as the use of *Wolbachia* to suppress vector population or prevent the transmission of dengue, with minimal social and environmental impact, are gaining attention and support worldwide[9, 12, 15, 22].

Despite having a rigorous vector surveillance and control program in Singapore, large-scale dengue epidemics in 2005, 2007 and 2013–2014, and the chikungunya outbreaks in 2008 and 2013 attests to the vulnerability of the country to outbreaks of mosquito-borne diseases. These epidemics also highlight the limitations of the current vector control strategies and reveal the need for a more innovative approach, such as the use of Wolbachia in tackling the challenges of dengue prevention and control in Singapore[23, 24]. In this study, we have examined the potential of the *w*Mel strain of *Wolbachia* when introgressed onto a Singaporean *Ae*. *aegypti* genetic background to interfere with arbovirus transmission. We have demonstrated that *w*Mel-Sg have significantly reduced ZIKV and CHIKV midgut infection and dissemination rates when compared to WT mosquitoes (Table 1). The titres of ZIKV and CHIKVs in *w*Mel-Sg were also significantly reduced (Figs 1 & 2). When midgut infection rates of ZIKV and CHIKVs in *w*Mel-Sg were compared, higher numbers of *Wolbachia*-carrying mosquitoes were found to be infected with ZIKV. However, the number of *w*Mel-Sg with disseminated infection, regardless of the infecting viruses, was very low.

Zika virus is currently emerging as a potential new arboviral threat that has resulted in the WHO declaring a global health emergency in Feb, 2015. The virus first gained attention when it caused a large-scale epidemic in the Pacific island of Yap, Federation of Micronesia in 2007 followed by outbreaks in French Polynesia, New Caledonia, the Cook Islands and Easter Islands in 2013–14 [2, 25–27]. In May 2015, the Ministry of Health Brazil confirmed its first case of ZIKV infection. Since then, the virus has spread to neighboring countries. Currently 26 countries in South America have reported autotochnous ZIKV transmission (ECDC, 2016). So far, outside of Africa, ZIKV has only been isolated from *Ae*. *aegypti* [28]. This study shows that *w*Mel is able to reduce ZIKV midgut infection and block the dissemination of the virus in *Ae*. *aegypti*. As such the current strategies being developed to use *Wolbachia* to control dengue will also simultaneously work on zika and chikungunya viruses.

The results from this study have shown that *w*Mel is able to block the dissemination of CHIKV and ZKV infection in *Ae*. *aegypti*’s salivary glands, the most important organ responsible for transmission. This indicates that establishment of *Wolbachia* in *Ae*. *aegypti* populations should reduce transmission of dengue, chikungunya and zika viruses and presents a potential control measure in this setting. It has also been proposed that releasing males may be an alternative way of using *Wolbachia* to suppress *Ae*. *aegypti* populations. A potential concern of this method is that incomplete sexing may result in safety concerns due to the release of female mosquitoes that could potentially contribute to virus transmission. The results of this study show that any inadvertently released female mosquitoes should have reduced vector competence and mitigate this risk.
